# Evaluative experiences: the epistemological significance of moral phenomenology

**DOI:** 10.1007/s11229-021-03044-4

**Published:** 2021-01-29

**Authors:** Philipp Berghofer

**Affiliations:** Department for Philosophy, University of Graz, Heinrichstraße 26/5, Graz, 8010 Austria

**Keywords:** Evaluative experience, Meta-ethics, Moral perception, Moral emotion, Phenomenology, Epistemology, Epistemic intuition, Myth of the given

## Abstract

Recently, a number of phenomenological approaches to experiential justification emerged according to which an experience's justificatory force is grounded in the experience’s distinctive *phenomenology*. The basic idea is that certain experiences exhibit a *presentive* phenomenology and that they are a source of immediate justification precisely by virtue of their presentive phenomenology. Such phenomenological approaches usually focus on perceptual experiences and mathematical intuitions. In this paper, I aim at a phenomenological approach to *ethical* experiences. I shall show that we need to make a distinction between evaluative experiences directed at concrete cases and ethical intuitions directed at general principles. The focus will be on evaluative experiences. I argue that evaluative experiences constitute a sui generis type of experience that gain their justificatory force by virtue of their presentive evaluative phenomenology. In Sect. 1, I introduce and motivate the phenomenological idea that certain experiences exhibit a justification-conferring phenomenology. In Sect. 4, I apply this idea to *morally evaluative experiences*. In Sect. 5, I suggest that certain *epistemic* intuitions should be considered *epistemically evaluative experiences* and I outline a strong parallelism between ethics and epistemology.

## Introducing the phenomenological approach to experiential justification

It is uncontroversial that experiences have a phenomenal character or phenomenology. An experience’s phenomenology denotes the what-it-is-like character to undergo the experience. For instance, when experiencing a black laptop, there is something it is like to undergo this black-laptop-experience. A black laptop is presented to you within experience and there is a clear phenomenal contrast between experiencing a black laptop and experiencing a red chair. Different experiences of the same type, e.g., visual experiences, typically differ phenomenologically. Of course, experiences of *different* types differ also phenomenologically (and even more drastically). In fact, as we shall see in Sect. 4, it makes sense to classify different types of experiences according to their respective distinctive type of phenomenology. We know that visual experiences differ phenomenologically from auditory or tactile experiences, perceptual experiences differ phenomenologically from introspective or imaginative experiences, etc. These phenomenological differences may be hard to pin down precisely, but by virtue of our first-person access to our experiential states we know they exist.

Furthermore, it is natural and plausible to assume that experiences have *justificatory force*. One’s perceptual experience as of a black laptop has justificatory force concerning the proposition that there is a black laptop.

Importantly, it should be uncontroversial that there is some relation between an experience's phenomenology and its justificatory force. My perceptual experience that presents to me a black laptop has justificatory force concerning the proposition that there is a black laptop; it does not have justificatory force concerning the proposition that there is a red chair behind me. When I turn around and experience the chair, my perceptual experience presenting a red chair has justificatory force concerning the proposition that there is a red chair; it does not have justificatory force concerning the proposition that there is a black laptop in the room.

What I call the *phenomenological conception of experiential justification* (PCEJ) is the claim that there is a straightforward relationship between an experience's phenomenology and its justificatory force. Regarding the question as to what it is that gives experiences their justificatory force, the phenomenological answer reads: Justification-conferring experiences gain their justificatory force precisely by virtue of their distinctive phenomenology. More formally, a strong version of PCEJ reads as follows:

An experience E provides immediate prima facie justification for believing a proposition *p if and only if* E has a justification-conferring phenomenology with respect to *p*.

Although this phenomenological approach seems to be the natural and commonsense approach to experiential justification, phenomenological approaches have not been popular in the analytic tradition. This is due to several reasons, including Sellars’ influential critique of the “myth of the given” and the dominance of externalist approaches. Recently, however, a number of promising and influential approaches emerged that qualify as phenomenological approaches (cf., e.g., Bengson 2015; Berghofer 2020a, b; Chudnoff 2013; Churchnoff 2013). Elijah Chudnoff framed the basic idea as follows, “the phenomenology grounds the epistemology” (Chudnoff 2016a, p. 117).

Phenomenological approaches have been introduced and defended particularly with regard to *perceptual* experiences (cf., e.g., Berghofer 2020a; Chudnoff 2018; Churchnoff 2013) and *mathematical intuitions* (cf., e.g., Berghofer 2020c; Chudnoff 2013, 2020). However, detailed phenomenological descriptions of and phenomenological approaches to the kind of experiences that are relevant in (meta-)ethics are still largely missing in the contemporary analytic literature. Such experiences include ethical intuitions, moral perceptions, and moral emotions. For the lack of a better term, I will subsume these experiences under the label “ethical experiences.” This paper is devoted to ethical experiences. I will show how to phenomenologically distinguish between the different types of ethical experiences, illuminate why such phenomenological distinctions are important, and suggest some novel parallels between meta-ethics and epistemology by arguing that certain epistemic intuitions should be regarded as evaluative experiences.

In the remainder of this section, I will briefly motivate the basic idea of the phenomenological conception: the claim that an experience’s justificatory force is grounded in its phenomenology.

### Degrees of givenness correspond to degrees of experiential justification

You can look at the same physical object from many different angles, different distances, under different light conditions, etc. All these experiences are directed at the same object, but they may well differ in how clearly and distinctly they present their object. These phenomenological distinctions (experiencing the object clearly and distinctly vs. vaguely and indistinctly) correspond to epistemic distinctions. A clear and distinct perceptual experience has more justificatory force than a vague and obscure one. In this sense, it is natural to assume that degrees of experiential justification correspond to degrees of givenness. Note that here we are exclusively discussing *experiential* justification. One’s background beliefs may undermine or support one’s experiential justification.

Of course, rival approaches to experiential justification may deny that degrees of justification correspond to degrees of givenness. A reliabilist, for instance, would insist that a vague and obscure experience can have more justificatory force than a clear and distinct one if the respective belief is more reliably produced by the former one. (For instance, in a scenario in which an evil demon systematically manipulates your surroundings such that your vague and obscure experiences are veridical but your clear and distinct ones are not.) It is not the aim of this paper to refute reliabilist or other rival accounts. Also, in this paper I do not play defense, defending phenomenological accounts against certain objections.^1^ Instead, in this section I only want to motivate PCEJ, showing that it is a plausible account that squares naturally with commonsense. The burden of proof should be on opponents of PCEJ.

### Contrasting presentive experiences with non-presentive experiences

One distinctive feature of perceptual experiences is that they have a *presentive* phenomenology. When you perceive the table in front of you, your experience does not merely represent the table, it *presents* the table to you. The table is given as bodily present. This is a clear phenomenal contrast to non-justification-conferring experiences and mental states. Thinking about, believing that, or imagining that there is a table does not justify you in believing that there is a table. These mental states lack the presentive phenomenology of perceptual experiences and they also lack their justificatory force. Arguably, it does not matter whether your wishful thinking that there is table reliably produces the belief that there is a table. Your wishful thinking that *p* can never (epistemically) justify you in believing that *p*. On the other hand, a clear and distinct perceptual experience, i.e., a perceptual experience with a pronounced presentive phenomenology, has justificatory force even if it turns out to be non-veridical or unreliable. This brings us to the following famous thought experiment.

### New evil demon problem

Imagine that person S and person S′ are undergoing perceptual experiences E and E′, respectively, such that E and E′ are descriptively or phenomenologically identical. This is to say that from the first-person perspective there is no difference between undergoing E and E′. Both present, say, there to be a red table. Now, we stipulate that E is veridical while E′ is non-veridical and even the product of an unreliable process. (An evil demon may systematically deceive S′.) Many epistemologists share the internalist intuition that in such a case E and E′ have the same justificatory force concerning the proposition that there is a red table. This implies that reliability is not necessary for justification. In this context, my point is simply that if we accept that E and E′ are equal in their justificatory force, then PCEJ provides the most natural and straightforward explanation. E and E′ are phenomenologically as well as justificatorily identical. The reason simply is that it is the phenomenology that grounds and determines the justificatory force. We may also spell this out in terms of a supervenience relation: An experience’s justificatory force supervenes on its phenomenology. Hence, if two experiences are alike phenomenologically, then they are alike justificatorily.

### Blindsight

Let us now turn to the real-world phenomenon of blindsight. We speak of blindsight if a person, due to a damaged visual cortex, suffers from conscious blindness but is nevertheless able to correctly respond to visual stimuli that the person is not consciously aware of. Often, the conscious blindness is restricted to a region of the person’s visual field, the person’s blind field. Experimental research shows that in certain scenarios a blindsight patient would report that she does not see anything that is going on in her blind field but when forced to guess, she correctly identifies the stimulus (cf. Lau 2008, p. 249).

Lawrence Weiskrantz who discovered and thoroughly investigated the phenomenon of blindsight defines blindsight as “'visual capacity in the *absence* of acknowledged awareness'” (Weiskrantz 1998, p. x). Obviously, such experimental research has important implications for the nature of visual consciousness and philosophy of mind. However, it has also significant *epistemological* implications. For instance, the phenomenon of blindsight puts pressure on reliabilist conceptions of perceptual justification (cf. Ghijsen 2016; Smithies 2014; Tucker 2010). While the new evil demon problem undermines the idea that reliability is *necessary* for justification, the phenomenon of blindsight undermines the idea that reliability is *sufficient* for justification. To see why consider the following hypothetical but empirically motivated example.

A blindsighted person S looks at a piece of sheet, where there is a circle in the region where S has normal sight (region R1) and a triangle at S′s blind field (region R2). Based on her perceptual experience, S believes that there is a circle in R1 and a triangle in R2. Are both beliefs equally justified? Even if we stipulate that the perceptual experience makes S strongly believe that there is a triangle at R2, there is an important *phenomenological* difference that corresponds to an *epistemological* difference.

In this example, S′s perceptual experience has a presentive character regarding the circle. S seems to be visually aware of the circle; the circle is presented to her within experience. This, however, is not true for the triangle. She might believe or be inclined to believe that there is a triangle, but it is not presented to her. This *phenomenological difference* fits perfectly with the *epistemological difference*. S′s perceptual experience provides her with *immediate* justification for believing that there is a circle in R1. Intuitively, however, S′s perceptual experience does not provide her with immediate justification that there is a triangle in R2. Of course, if S knows that her blindsight faculties are reliable in the sense that in the past most of her blindsight based beliefs have turned out to be true, then S may be inferentially justified in believing that there is a triangle. But such justification cannot be immediate. A plausible conception of perceptual justification should be able to avoid the consequence that blindsight experiences can be a source of immediate justification (cf. Ghijsen 2016, pp. 17–19 and Smithies 2014, p. 103f.).

What is often overlooked is that well-known examples such as the new evil demon problem and the phenomenon of blindsight not only play a negative function in undermining certain approaches but can also have a *positive* function, *motivating the close connection between epistemology and philosophy of mind*. What these examples suggest is that epistemology is grounded in phenomenology. This is why descriptive/phenomenological investigations of how different (types of) experiences present their respective objects and contents are of great epistemological significance. This paper focuses on *moral* phenomenology, addressing the phenomenal character of *evaluative* experiences. Before I turn to this main topic of the paper, I shall discuss a Sellarsian-style anti-foundationalist criticism of PCEJ.

## A Sellarsian-style anti-foundationalist criticism of PCEJ

Concerning Sect. 1, an anonymous referee of this journal raised a “Sellarsian-style anti-foundationalist criticism of PCEJ.” This criticism is directed against the claim that the distinctive presentive phenomenology of an experience can be *sufficient* for experiential justification (which is implied by PCEJ). The idea of this criticism is that “the depth or richness of what a given experience justifies us in believing depends on our background knowledge/competence (in addition to the relevant phenomenological qualities of the experience).” A similar worry can be found in Sellars when he states that proponents of the given “have taken givenness to be a fact which presupposes no learning, no forming of associations, no setting up of stimulus–response connections” (Sellars 1997, p. 20). Instead, Sellars emphasizes that “all knowledge *that something is thus-and-so* […] involves learning, concept formation, even the use of symbols” (Sellars 1997, p. 20). The anonymous referee exemplifies this by specifying the following example: “A particular visual experience might justify me in believing that I see a bird; the same experience might justify an ornithologist or bird watcher in believing that she sees a red-bellied woodpecker.”

By stipulating that it is the *same*^2^ experience that justifies the expert but not the novice in believing a certain proposition, this would refute my claim that an experience's justificatory force *supervenes* on its phenomenology. It is to be noted that this criticism rests on two highly controversial premises:

- P1: Both experiences have the same phenomenology.
- P2: The experience provides the ornithologist with immediate justification that this bird is a red-bellied woodpecker.

There are several reasons why one might doubt P2. For instance, one might insist that perceptual experiences can only represent low-level properties, such as colors and shapes, and that the expert’s justification is *inferential* justification. Importantly, even if you (i) believe that perceptual experiences can *represent* high-level properties such as “being a red-bellied woodpecker” and (ii) subscribe to PCEJ, you may still reject P2 if you believe that perceptual experiences fail to have a *presentive* phenomenology with respect to such high-level properties. For such an account, cf. Chudnoff (2018). In what follows, however, I will briefly elaborate on why I reject P1.

I take it that the case described by the referee is a case of *perceptual learning*. Perceptual learning, broadly speaking, “refers to an increase in the ability to extract information from the environment, as a result of experience and practice with stimulation coming from it” (Gibson 1969, p. 3). Such examples of perceptual learning have become increasingly popular in current philosophy of mind. In particular, perceptual learning has been a focus of the works of Susanna Siegel and Kevin Connolly. Siegel discusses the hypothetical example in which one has never seen a pine tree before and gets hired to cut down pine trees (cf. Siegel 2010, p. 100). After several weeks, one is able to identify pine trees on sight and distinguish them visually from other trees. According to Siegel, one’s experiences of pine trees before and one’s experiences of pine trees after perceptual learning has taken place differ *phenomenologically* (cf. Siegel 2010, p. 101). Connolly discusses the example of an expert birdwatcher who is looking at a wren. Connolly is in agreement with Siegel when he states that “the perception of an expert birdwatcher is phenomenally different from the perception of a layperson, even when viewed under the exact same background conditions” (Connolly 2014, p. 2). In this light, it makes sense to reject the referee’s premise that the expert and the novice are undergoing the same perceptual experiences. They do not because their experiences differ phenomenologically. Perceptual learning has affected how the expert perceives birds.

It is to be noted that there is no universal agreement that perceptual learning involves phenomenal changes since there are scattered examples in the literature in which putative cases of perceptual learning are approached as changes in judgments/beliefs instead of changes in the experience’s phenomenology. The most extensive and convincing defense of the claim that perceptual learning is genuinely perceptual and involves phenomenal changes is offered in (Connolly 2019, Chapter 2). Here Connolly elucidates “converging evidence that comes from different levels of analysis: from philosophical introspection, neuroscience, and psychology” (Connolly 2019, p. 46). Concerning philosophical introspection, Connolly invokes the “multiplicity of philosophers from different times and places who independently argue, based on introspection” that perceptual learning involves perceptual changes. Regarding neuroscience, Connolly discusses the “neuroscientific evidence that perceptual learning modifies the primary sensory cortices” (Connolly 2019, p. 48), arguing that this is why most scientists do indeed consider perceptual learning a genuinely perceptual process.

I take Connolly to have successfully shown that perceptual learning should be considered a process that involves phenomenal changes. Furthermore, I wish to emphasize that from a phenomenological point of view, it is only natural to assume that different people can perceive the same object very differently. This is because perceptual experiences are genuinely perspectival, they are shaped by previous experiences as well as our beliefs and expectations, and they are affected by the concepts we use (cf. Berghofer 2020d).

This also shows that Sellars’ worry that proponents of the given “have taken givenness to be a fact which presupposes no learning, no forming of associations, no setting up of stimulus–response connections” (Sellars 1997, p. 20) does not apply to my phenomenological account of givenness. One might wonder, then, how could experiences, given that they are shaped by beliefs, concepts, and previous experiences, be a source of immediate justification? The key to answering this question is to be found in James Pryor’s defense of immediate justification (Pryor 2000, 2005). Proponents of immediate justification do not have to deny that entertaining basic beliefs (i.e., immediately justified beliefs) is only possible if you have certain faculties, concepts, empirical input, etc. beforehand. Perhaps all our beliefs depend on other beliefs for their *existence*, but some beliefs do not depend on other beliefs for their *justification*.

The fact that you have immediate justification to believe P does not entail that no other beliefs are required for you to be able *to form or entertain* the belief that P. Having the concepts involved in the belief that P may require believing certain other propositions; it does not follow that any justification you have to believe P must be mediated by those other propositions. (Pryor 2005, p. 183).

In terms of *experiences*, we say that various factors such as background beliefs, learning, and concept formation may play a role in why a certain experience has a presentive phenomenology precisely with respect to *p*. This may imply that the experience and perhaps the respective belief that *p* depend on other beliefs for their existence; but it does not imply that the belief that *p* depends on other beliefs for its justification. So how can perceptual experience be a source of immediate justification despite involving processes of learning and concept formation? My answer is straightforward: All depends on the experience’s phenomenology. If a perceptual experience presents a table being in front of you, you are immediately justified in believing that there is a table in front of you, simply because your experience has a “presentive” phenomenology with respect to this object/content. Concerning the experience’s justificatory force: It only matters that the experience has a presentive phenomenology; it does not matter *why* the experience has such a phenomenology.

However, an experience’s justificatory force can be undermined (or supported) by one’s background beliefs. An anonymous referee of this journal asked me to clarify the relationship between experiential justification and background beliefs. In this context, the referee raised the following questions:

“Is experiential justification something that accrues independently of background belief, which can then either defeat or bolster the justification which the belief (justified by this experience) has? Or does the background belief bear directly on the degree of experiential justification in question?”.

I answer the first question in the affirmative and the second one in the negative. Experiential justification is epistemically independent of background beliefs and although background beliefs can defeat or support experiential justification, they cannot bear directly on the degree of experiential justification.^3^ Examples of background beliefs defeating experiential justification would be cases of known illusions such as the Müller-Lyer illusion. (In this case, your experience provides you with immediate prima facie justification for believing that the lines differ in length, but this experiential justification is defeated by what you know about Müller-Lyer illusions.) Examples of background beliefs supporting experiential justification are everyday cases in which what you experience coheres with what you believe and expect.

Having clarified the details of my phenomenological account of experiential justification, I will now apply it to ethical experiences.

## Ethical experiences: Phenomenological distinctions

Consider the following four scenarios:

- S1: You are walking down the street when you witness a group of hoodlums burning a cat (Harman 1977, p. 4). The cat is obviously suffering. The hoodlums are laughing. You are appalled and disgusted and consider the action of burning the cat as morally wrong.
- S2: You are reading *Harry Potter*, vividly imagining how Harry is mistreated and abused by his adoptive family, the Dursleys. You hate the Dursleys for how they treat Harry.
- S3: You are contemplating the ethical principle that there is a prima facie duty not to torture.
- S4: You are contemplating the meta-ethical principle that moral properties supervene on natural properties.

In the literature, ethical intuitionism is understood as the doctrine “that some of our ethical knowledge is non-inferential” (Väyrynen 2008, p. 489; cf. also Audi 2007, p. 201). Accordingly, ethical intuitions are often considered to be mere beliefs (Audi 2004, p. 34). I shall argue that at least some so-called ethical intuitions should be understood as *experiences* and that current debates would benefit from more detailed *phenomenological analyses* of how such ethical experiences (re-)present their respective contents/objects. More precisely, in this section, I wish to clarify that there are significant phenomenological differences between the kind of ethical experiences that are relevant in S1 and S2, on the one hand, and the ones that are relevant in S3 and S4, on the other hand. In the literature, there is no agreement about the nature of the ethical experiences that play a role in such cases or even whether there are ethical experiences. Regarding S1, for instance, we can distinguish between the following approaches.

1. *Inferential justification* There are no ethical experiences. Your experiences do not have any evaluative contents. You experience only what is going on physically and your experiences are a source of immediate justification only with respect to the represented physical contents. You experience the hoodlums burning the cat and thereby you are justified in believing that the hoodlums are burning the cat. Your belief that this action is morally wrong is epistemically dependent on your moral background beliefs such as that causing pain for fun is morally wrong. The inferential account has been expressed by Nicholas Sturgeon as follows: “Given my broad understanding of inference, moreover, I take this to mean that moral observation, even when it doesn’t require stopping to ‘figure anything out’, always does involve inference, automatic and unconscious, and that among the premises are moral views one already has.” (Sturgeon 2002, p. 205)
2. *Moral perception* Your perceptual experience not only (re-)presents physical properties but also moral properties. Similar to how you can see that the table in front of you is black, you can see that the action of burning the cat is morally wrong. The wrongness of the action is part of the content of your perceptual experience. What we here call moral perception has been referred to as “Canonical Evaluative Perception” (Bergqvist & Cowan 2018, p. 5) or “Contentful Moral Perception” (Werner 2020, p. 5). Proponents include Audi 2013, Cowan 2015, and Werner 2016. In the literature, we also find weaker accounts of moral perception such as the idea that moral properties are patterns and moral perception is pattern-recognition (Chappell 2008) or Sarah McGrath’s account according to which perceptual experiences do not have normative contents but can still immediately justify moral beliefs (McGrath 2018).
3. *Moral emotion* By perceiving the scene you are undergoing an emotional experience. Emotional experiences have evaluative contents. The action of burning the cat is (re-)presented, e.g., as despicable. The idea here is that emotional experiences are epistemically as well as metaphysically similar to perceptual experiences. They are experiences that represent certain properties such that they are a source of immediate justification concerning their contents. The claim that emotional experiences can immediately justify moral beliefs is championed, e.g., by Mitchell 2017 and Pelser 2014. Emotions are not only similar to perceptions, perceptions can serve as the basis of an emotion. In our case of the hoodlums burning the cat, you emote about something you perceive. Sometimes moral emotions are considered moral perceptions (cf. Werner 2020, p. 6).
4. *Sui generis experience* Your evaluative experience that (re-)presents the action of burning the cat as morally wrong or despicable can not be reduced to a perceptual, emotional, or any other kind of experience but is sui generis. However, this evaluative experience may be “an amalgam of sensory, emotional, and imaginative components” (Bergqvist and Cowan 2018, p. 5).
5. *Intellectual seeming* According to a popular approach, the experience in question is an a priori intuition, namely a moral intellectual seeming. It seems to you that burning the cat is morally wrong. This seeming is not a perceptual experience but an a priori intellectual seeming. Just like a *perceptual* seeming can justify you in believing that there is a table, a *moral intellectual* seeming can justify you in believing that an action is morally wrong/good.

I believe that A1 is mistaken. It is not the case that you only experience what is going on physically and infer that the action in question is morally wrong. Instead, you experience the action as morally wrong or despicable. But what type of experience is it that presents the action as morally wrong? Is it a moral perception, emotion, an intellectual seeming, or something else? I believe that the experience in question is best characterized as an *evaluative experience*, i.e., an experience whose distinctive phenomenology consists in presenting its object in an evaluative manner. In Sect. 4, I will argue that such evaluative experiences are best understood as a sui generis type of experience. This means that I side with option 4. However, I will also point out that evaluative experiences may be integrated into, e.g., perceptual experiences such that one may stick to the terminology of moral perceptions, emotions, or seemings. Importantly, while my approach may be consistent even with option 5, I believe that the seeming-terminology has some severe shortcomings in that it obfuscates the significance of phenomenological analyses. Let me explain.

In current debates, the seeming-terminology has been mainly coined by Michael Huemer and his principle of phenomenal conservatism (PC). PC offers a unified and straightforward account according to which prima facie justification for beliefs about the external world, the inner world, mathematics, ethics, and so forth is easily to be gained:

PC: “If it seems to *S* as if *P*, then *S* thereby has at least prima facie justification for believing that *P*.” (Huemer 2001, p. 99).

Huemer's PC has become very popular. But what is a seeming? Phenomenal conservatives typically regard seemings as sui generis propositional mental states (cf., e.g., Huemer 2007 and Tucker 2010). Sui generis means that seemings are irreducible. In particular, they cannot be reduced to beliefs or inclinations to believe. Tucker calls this the experience-view: “A seeming that P is: […] An experience with the content P or a sui generis propositional attitude that P” (Tucker 2013, p. 3).

The most distinctive feature of seemings is that they present their contents as true. “The real difference between seemings and other states that can incline one to believe their contents is that seemings have the feel of truth, the feel of a state whose content reveals how things really are” (Tolhurst 1998, p. 298f.). In this context, Huemer speaks of the “forcefulness” of seemings, whereas Tucker prefers the term “assertiveness.” Tucker offers the following definition of a seeming's phenomenology: “The phenomenology of a seeming makes it feel as though the seeming is 'recommending' its propositional content as true or 'assuring' us of the content's truth.” (Tucker 2010, p. 530).

Obviously, Huemer’s PC has much in common with PCEJ. Both emphasize the justificatory force of experiences and ascribe a distinctive phenomenology to the respective justification-conferring experiences.

What distinguishes PC from PCEJ, the reason why PC does not amount to a version of PCEJ, is that PC does not state that seemings are justifiers *because of their phenomenology*. For Huemer, being a seeming is sufficient for having justificatory force, but he does not hold that a seeming has justificatory force *qua* having this distinctive seeming-phenomenology. Accordingly, PC does not provide an answer to our question of what it is that makes certain experiences a source of justification. PC implies that certain experiences are justifiers insofar as they are seemings, but it does not clarify why seemings are justifiers in the first place. More importantly, I believe that the phenomenological characterization of “making it seem to one that *p*” (i) does not do justice to the diversity of justification-conferring experiences and thus fails to adequately capture the distinctive phenomenology of perceptual (and other justification-conferring) experiences and (ii) is too vague and thereby leads to well-known counterexamples to PC (cf. Markie 2005 and Berghofer 2020a).

Let me exemplify the criticism expressed by (i) by revisiting the four scenarios specified at the beginning of this section. A phenomenal conservative may say that in all four cases one is justified in believing the respective propositions simply because, e.g., it seems to one that the action of burning the cat is morally wrong or because it seems to one that there is a prima facie duty not to torture. Of course, this approach has the virtue of providing a consistent and unified account. However, phenomenologically, there is much more to say about the respective experiences. And if my phenomenological approach outlined in Sect. 1 is on the right track, these phenomenological distinctions are epistemologically significant. What is more, if the popular objection is correct that PC is too liberal in granting any seeming justificatory force, we are in urgent need for a more adequate and specific phenomenological characterization.

This is to say that what we need is a more careful *moral phenomenology*. “Moral phenomenology” either denotes (i) the phenomenology of an ethical experience or (ii) the descriptive study of the phenomenology of ethical experiences. Here I use it in the sense of (ii). In current analytically dominated debates, moral phenomenology has not played an important role.^4^ Any descriptive, phenomenological analysis requires a twofold specification. First, what are the objects/contents the respective experiences are intentionally directed at? Second, how do they present their objects/contents? Considering the four scenarios S1–S4, we can easily make the following distinctions.

**Table:** 

(1)	While S1 and S2 deal with experiences that are directed at concrete real/hypothetical cases, S3 and S4 deal with experiences that are directed at general principles
(2)	While the experiences in S1 and S2 present their objects in an evaluative manner, the experiences in S3 and S4 present their objects as necessarily true

The idea is that the *evaluative* experiences in S1 and S2 are closer to perceptual experiences. Similar to how I can see that the table in front of me is black, I can experience an action to be morally wrong/good. The (meta-)ethical intuitions in S3 and S4, on the other hand, are closer to mathematical intuitions. Similar to how I can intuit that the number two is the only even prime number, I can intuit that there is no scenario in which torturing is prima facie good. Of course, there is much more we need to say about the phenomenology of these experiences. In the following sections, I focus on evaluative experiences.

## Evaluative experiences

In Sect. 1, I have introduced and motivated PCEJ, i.e., the phenomenological idea that certain experiences gain their justificatory force by virtue of their distinctive phenomenology. For our purposes, the most relevant implication of PCEJ is that an experience is a source of immediate justification if and only if it exhibits a distinctive presentive phenomenology. In Sect. 3, I have contrasted different types of ethical experiences. I distinguished between ethical experiences that are directed at concrete (real or hypothetical) scenarios, presenting their objects/contents in an evaluative manner and ethical intuitions that are directed at general principles, presenting their objects/contents as necessarily true. In what follows, I focus on the former type of experiences. I call them evaluative experiences.

An example of an evaluative experience is when you witness hoodlums burning a cat and experience this action as despicable and morally wrong. As discussed in the previous section, one prominent approach is to say that this experience is a moral perception and that you can literally see that this action is morally wrong. However, there are a number of prominent objections to the possibility of moral perception. Many of these objections concern the nature of perception.

For instance, there is the worry that has been termed the “causal objection.” This objection rests on the assumptions that (i) perception is a causal process and that (ii) moral properties are causally inert, concluding that moral properties cannot be represented in perception.^5^ Similarly, prominent voices deny that high-level properties can be represented in perceptual experiences. Since moral properties are high-level properties (in contrast to low-level properties such as color and shape), it follows that moral properties cannot be represented in perception.^6^ My phenomenological account avoids such objections. As I will elaborate in more detail below, it simply does not matter whether the evaluative experience is integrated into a perceptual experience. All that matters is whether the evaluative experience exhibits a distinctive presentive character.

Regarding evaluative experiences, a phenomenological moral epistemology must address two questions in particular:

- Q1: Do we experience certain concrete cases in a distinctively morally evaluative manner?
- Q2: If so, does this evaluative phenomenology qualify as a presentive phenomenology?

Q1 and Q2 can be reformulated as asking: Are there morally evaluative experiences, and, if so, do they possess a presentive phenomenology? If both questions are answered affirmatively, it follows from our phenomenological account outlined in Sect. 1 that morally evaluative experiences are a source of immediate justification. For a moral epistemology, this means that *one* way of gaining moral knowledge is via evaluative experiences of concrete cases.^7^

In this section, I argue that Q1 must be answered affirmatively and that the answer to this question does not hinge on the question of whether moral perception is possible. This can be shown most straightforwardly by contrasting evaluative experiences directed at concrete *real* cases with evaluative experiences directed at concrete *hypothetical* cases. To put it differently, I contrast moral perceptions^8^ with moral imaginations.

Recall Harman’s example of hoodlums burning a cat we discussed in the previous section. The point of this example is that when witnessing this action, you gain moral knowledge that this action is morally wrong and one explanation for this knowledge is that you literally perceive this action to be morally wrong. However, you do *not* need to actually *perceive* this action to gain moral knowledge. When *imagining* this hypothetical scenario, you also gain moral knowledge. You gain the insight that it would be morally wrong to burn the cat. One explanation for this knowledge is that you experience this hypothetical action as morally wrong and that this evaluative experience is a source of immediate justification.

Let us contrast the two cases (perceived real case vs. imagined hypothetical case) in more detail. What are the phenomenological distinctions and similarities? In both cases, you are intentionally directed at the concrete action of burning a cat. In the real case, you visually experience the hoodlums burning a cat. In the hypothetical case, you imagine the hoodlums burning a cat. In the real case, there may be a variety of *affective*, *emotional* responses, such as anger, fear, and disgust, that accompany your perceptual experience. Furthermore, there may be a variety of *bodily responses* such as a tension of your muscles, goosebumps, or being on the verge of tears. In the hypothetical case, there may be similar emotional and bodily responses. Perhaps not in this well-known and often-discussed example, but pieces of literature can evoke such reactions. When Harry is mistreated by his adoptive family in *Harry Potter* or when a beloved character is beheaded or tortured in *A Song of Ice and Fire* the reader shows emotional responses such as anger and sadness and often also bodily responses such as goosebumps or tears. More importantly, in both cases, there is an evaluative component involved. When you perceive as well as when you imagine the action of burning the cat, this action is experienced as morally wrong.

This evaluative component is particularly obvious in the presence of strong emotional responses. In fact, in the contemporary literature emotions are characterized as evaluative experiences. “It is now widely accepted that emotions present their object (their ‘intentional object’) in a certain evaluative way” (Cova et al. 2015, p. 397). In this terminology, moral emotions are experiences that present their object in a morally evaluative way. Importantly, such emotional experiences that present their contents in an evaluative manner are possible with respect to real cases as well as with respect to hypothetical cases. Even more importantly, it is precisely this *evaluative phenomenology* that constitutes the distinctive presentive character of moral perceptions and moral imaginations. This is to say that if moral perceptions and moral imaginations are a source of immediate justification, they are so by virtue of their evaluative phenomenology. I say an experience exhibits an evaluative phenomenology if it presents its objects/contents in an evaluative manner.

One may object that there is no significant phenomenal difference between an evaluative *judgment* and what I call an evaluative *experience*. I submit that there is a phenomenal difference that is similar in kind to the difference between *believing* that there is a table in the next room and actually *visually experiencing* the table. You may have overwhelming non-perceptual evidence that there is a table in the room next to you. For instance, a number of people you know to be reliable just told you. Your belief that there is a table in the next room is justified but it is *inferentially* justified. However, when you go and check and are perceptually aware of the table, your table-experience presents the table to you and provides you with *immediate* prima facie justification for believing that there is a table. Similar stories can be told concerning the phenomenal and epistemic differences between evaluative beliefs/judgments and evaluative experiences.

Let us begin with an example from aesthetics. Say, somebody you know to be a reliable art critic tells you that Van Gogh's *The Starry Night* is a beautiful painting. You also know that the painting exemplifies a post-impressionist style and color composition that you deem to be beautiful. Accordingly, you might form the (inferentially justified) belief “*The Starry Night* is beautiful.” This is an evaluative belief that might be accompanied by passive feelings. Neither this evaluative belief nor these passive feelings are justifiers. Neither this belief nor these passive feelings exhibit an evaluative phenomenology. They do not present the painting as beautiful. Now assume you visit the Museum of Modern Art (MoMA) and take a look at the painting, experiencing the painting as beautiful. Now you are undergoing an evaluative experience that provides you with immediate justification for believing that the painting is beautiful.

Analogously, there are phenomenal and epistemic differences between morally evaluative *beliefs* and *experiences*. When reading about Harman’s example of hoodlums burning a cat, you may be like “Sure, inflicting pain on a sentient being is prima facie wrong, so burning a cat for fun is prima facie wrong”. This evaluative judgment is *inferentially justified*. However, when you actually witness a cat being burnt to death in front of you (or imagine this scenario vividly), you should be undergoing an evaluative experience that *presents* this action as morally wrong and despicable. This evaluative experience provides you with immediate prima facie justification for believing that the action of burning a cat is morally wrong.

Let me summarize the theses I aimed at establishing in this section:

- T1: Evaluative experiences exist. Sometimes we experience concrete cases in a distinctively evaluative manner.
- T2: Evaluative experiences possess a justification-conferring presentive phenomenology. This is to say that evaluative experiences are a source of immediate justification and that they gain their justificatory force precisely by virtue of their distinctive presentive evaluative phenomenology.
- T3: Evaluative experiences can occur with respect to real but also with respect to hypothetical concrete cases.
- T4: Evaluative experiences are sui generis experiences that cannot be reduced to perceptual or imaginative experiences but that can emerge from perceiving or imagining concrete cases.

What is novel and original about my paper concerns particularly T2 and T4. Concerning T2, although it is sometimes mentioned in the literature that the internalist may argue that ethical experiences such as moral emotions justify by virtue of their phenomenology (cf., e.g., Cowan 2018, p. 223), I am not aware of any work that actually provides a detailed phenomenological account, arguing that certain ethical experiences possess a presentive evaluative phenomenology such that they justify by virtue of their presentive evaluative phenomenology.^9^ The virtue of my phenomenological account is that moral epistemology becomes deeply embedded in the underlying phenomenological epistemology. (Such as how the present section draws on the results of Sect. 1.) Proponents of moral perception often mainly “play defense,” reacting to objections that deny that moral properties could be represented in perception. My phenomenological account allows for an offensive strategy. If evaluative experiences possess a distinctive evaluative phenomenology, it is natural to consider them sources of immediate justification.

T4 is motivated by our result that evaluative experiences can occur with respect to *perceived real* cases but also with respect to *imagined hypothetical* cases.^10^ Accordingly, it is plausible to assume that they cannot be reduced to perceptual or imaginative experiences. Furthermore, since evaluative experiences possess a distinctive evaluative phenomenology, it is natural from our phenomenological perspective to classify them according to their phenomenology. Perceptual experiences are experiences that exhibit a distinctive perceptual phenomenology; imaginative experiences are experiences that exhibit a distinctive imaginative phenomenology; and evaluative experiences are experiences that exhibit a distinctive evaluative phenomenology.

As pointed out at the beginning of this section, this result that evaluative experiences are sui generis experiences that cannot be reduced to perceptual experiences has important implications for moral epistemology. This is because in the literature there are prominent objections to the claim that moral properties can be represented in perception. But how is immediate moral knowledge about concrete cases possible if perceptual experiences cannot justify moral beliefs? The answer is by way of evaluative experiences. To be sure, I do not deny the possibility of moral perception. Evaluative experiences may be integrated into perception such that it makes sense to speak of moral perception. The point is it does not matter. What matters is that there are good reasons to believe that evaluative experiences exist and that evaluative experiences have a presentive phenomenology, which—if my phenomenological epistemology as outlined in Sect. 1 is correct–means that evaluative experiences can non-inferentially justify evaluative beliefs.^11^

I wish to conclude this section by addressing the relationship between evaluative experiences and background beliefs. Here I apply my account specified in Sect. 2. This means that I propose that if an experience exhibits a presentive evaluative phenomenology with respect to *p*, having such a presentive phenomenology is *sufficient* for the experience to provide prima facie justification for believing that *p*. As specified in Sect. 2, background beliefs can undermine or support experiential justification. For instance, your experience may present to you a specific action as improper, but you might know that because of your overly religious and conservative upbringing you are biased with respect to this type of action. This background belief, then, can defeat your experiential justification. This would be similar to the cases of known illusions discussed in Sect. 2. Of course, this leaves open many important questions concerning the relationship between experiential justification and background beliefs. But since this paper is concerned with experiential justification, I shall leave it at that.

In the following final section, I suggest that evaluative experiences play a more substantial role in epistemology than is commonly assumed. This is because certain epistemic intuitions may better be understood as epistemically evaluative experiences.

## Epistemic intuitions as evaluative experiences?

Intuitions have always been of central philosophical interest. From Plato to Augustine, to Descartes, to Kant, to Husserl, for all these thinkers the nature and epistemic role of intuitions were a central theme of their philosophical investigations. The significance of intuitions may be particularly obvious in current analytic philosophy. This is because regarding orthodox philosophical methodology, there is considerable agreement that “intuitions are presented as our evidence in philosophy” (Williamson 2007, p. 214) and that “analytic philosophy without intuitions just wouldn’t be analytic philosophy” (Weinberg 2007, p. 318; cf. also Pust 2000, p. xiii). The reliance on intuition is considered as one of the defining features of philosophy: “One thing that distinguishes philosophical methodology from the methodology of the sciences is its extensive and avowed reliance on intuition” (Goldman 2007, p. 1).^12^

Of particular importance in contemporary philosophy are so-called epistemic intuitions such as the famous Gettier intuitions. In a loose sense, epistemic intuitions are understood as “immediate assessments arising when someone’s condition appears to fall on one side or the other of some significant divide in epistemology” (Nagel 2007, p. 792) or as “a label for any immediate (or not explicitly inferential) assessment of any claim of interest to epistemologists” (Nagel 2007, p. 793). Analogously to how we proceeded with respect to ethical experiences above, I submit that at least some so-called epistemic intuitions should be understood as *experiences* and that current debates would benefit from more detailed *phenomenological analyses* of how such epistemic experiences (re-)present their respective contents/objects.

What I suggest in this final section is that, analogously to moral experiences, epistemic experiences can be distinguished in epistemically evaluative experiences directed at concrete cases and epistemic intuitions directed at general principles. Interestingly, to my knowledge, neither in the analytic nor in the phenomenological traditions there are elaborate accounts of epistemically evaluative experiences. This is surprising since in both traditions there is consensus that epistemic values exist (cf., e.g., Haddock et al. 2009 and Husserl 1996, p. 294).^13^ If there are epistemic values and if we know them to exist, from a phenomenological perspective it is only natural to assume that we have some experiential access—at least in the modest sense that certain objects are presented to us in an epistemically evaluative way.

Let us now turn to some propositions that are typically considered prime examples of contents of epistemic experiences/intuitions:

- I1: In Gettier’s famous thought experiment, Smith has a justified true belief that the person who will get the job has ten coins in his pockets, but Smith does not know that the person who will get the job has ten coins in his pockets.
- I2: When Neo lives in the Matrix and has a clear and distinct perceptual experience as of a desk, he is justified in believing that there is a desk.
- I3: It is possible that a person has a justified true belief that *p*, but does not know that *p*.
- I4: Perceptual experiences are a source of prima facie justification.

As a side note, it is important not to confuse an experience with its content. I3, for instance, is not an intuition but a proposition (expressing a general principle) one may be able to intuit. This is analogous to how a desk is not a perceptual experience but the kind of object that can be perceived. Furthermore, as discussed above, different mental states can be directed at the same object, can have the same content. I can believe that there is a desk in the next room or I can go and look. I may believe that 2 is the only even prime number because I have read it in a textbook or I can contemplate this statement and after some time “see” why it must be true. Similarly, I may believe that I3 holds because I know that most epistemologists agree, or I may be able to *intuit* it in a sense to be specified.

We note that I3 and I4 express *general principles* while I1 and I2 are statements about *concrete scenarios*. I argue that, analogously to the case of ethical experiences, there is a *significant phenomenal contrast* between intuiting a principle like I4 and being intentionally directed at a concrete scenario such as in I2.

Concerning I1–I4, I suggest the following distinctions.

**Table:** 

(1)	While I1 and I2 deal with experiences that are directed at concrete (but hypothetical) cases, I3 and I4 deal with experiences that are directed at general principles
(2)	While the experiences in I1 and I2 present their objects in an evaluative manner, the experiences in I3 and I4 present their objects as necessarily true

The idea is that the *epistemically evaluative* experiences in I1 and I2 are phenomenologically similar to morally evaluative experiences. Similar to how I can experience the act of burning a cat as morally wrong, I can experience a belief to be epistemically (un-)justified. The epistemic intuitions in I3 and I4, on the other hand, are analogous to ethical intuitions of general principles. Similar to how I can intuit that there is a prima facie duty not to torture, I can intuit that perceptual experiences are a source of prima facie justification.

One difference, of course, between morally evaluative experiences and epistemically evaluative experiences concerns the objects they are directed at. While the former are primarily concerned with (human) *actions*, e.g., the hoodlums burning the cat, the latter are primarily concerned with (human) *beliefs*. Furthermore, while morally evaluative experiences are typically accompanied by or integrated into emotional responses, epistemically evaluative experiences usually are not.

Consider the following scenario:

Sarah and Vincent are looking at an apple tree. By visually undergoing this experience, a tree with green leaves and red apples is presented to them. Based on this experience, Sarah forms the belief that there is a tree with green leaves and red apples. Based on this experience, Vincent forms the belief that the tree is the incarnation of his deceased dog.

Considering this example, you may form the judgments that, by undergoing this perceptual experience, Sarah’s belief is justified and that Vincent’s belief is not. I suggest that these *evaluative* judgments can be non-inferentially justified by evaluative experiences presenting to you Sarah’s belief as justified and Vincent’s belief as unjustified.

Of course, there are also other ways your judgments could be justified. For instance, you may subscribe to PCEJ, believing that a perceptual experience E can immediately justify believing a proposition *p*, only if E has a presentive perceptual phenomenology with respect to *p*. Since the proposition that the tree is the incarnation of Vincent’s dog is not presented to Vincent within experience, Vincent is not experientially justified in believing that the tree is the incarnation of his dog. If this is your line of reasoning, your judgment about the epistemic status of Vincent’s belief is *inferentially* justified. This is analogous to how your judgment that burning the cat is morally wrong can be justified immediately by an evaluative experience or inferentially by inferring it from underlying moral principles. However, while in the moral case your evaluative experience may be accompanied by emotional responses such as anger or disgust, Vincent forming an unjustified belief likely will not affect you emotionally.

This being said, I believe that sometimes epistemically evaluative experiences are accompanied by strong emotions such that we may speak of *epistemic emotions* (analogously to the established concept of moral emotions). Assume, a relative tells you that he now believes that the COVID-19 pandemic was caused by the Democratic Party to compromise Trump’s reelection plans and that the virus is only a mild flu such that no restrictions are needed to prevent its further spread. He believes so based on a YouTube video he recently saw. You may regard your relative’s beliefs as unjustified, lacking evidence, biased, inconsistent, or stupid. My point is that these are *evaluative* judgments, namely *epistemically* evaluative judgments. The idea is that such an evaluative judgment can be based on an evaluative experience that presents your relative's belief as stupid similar to how your judgment that the hoodlums burning the cat can be based on your evaluative experience that presents this action as despicable. If the picture sketched in this section is correct, this would imply a profound parallelism between ethics and epistemology.

## Conclusion

According to the proposed phenomenological approach to ethical and epistemic experiences, we need to make a distinction between evaluative experiences directed at concrete cases and intuitions directed at general principles. The focus was on evaluative experiences which I argued to be a sui generis type of experience. The idea is that such evaluative experiences are a source of immediate justification concerning evaluative beliefs and that they gain their justificatory force precisely by virtue of their distinctive evaluative phenomenology. In the final section, based on the conception of evaluative experience, I suggested that there is a striking parallelism between ethics and epistemology.

## Footnotes

1 For a defense of PCEJ against a recent objection, cf. Berghofer (2020b), Sect. 4.

2 I take it that “same” here means that both experiences have the same phenomenology, i.e., phenomenal character.

3 The only exception to this rule would be cases of cognitive penetration in which one’s background beliefs affect the presentive phenomenology of an experience. If such cases are possible, this would make the relationship between experiences and beliefs more complicated, but such cases would not pose any specific problem for my PCEJ.

4 Of course, there are notable exceptions. For works that either (not necessarily explicitly) engage in or at least argue for the significance of moral phenomenology, cf., (e.g., Audi 2013; Chudnoff 2016b; Chudnoff 2013; Milona 2018; Werner 2020).

5 For a defense of moral perception against the causal objection, cf. McBrayer 2010. For a summary of this discussion, cf. Werner (2020).

6 For a discussion of this problem, cf. Milona (2018), whose line of reasoning is similar to mine.

7 Another way may be via ethical intuitions concerning general principles.

8 I am now using the term “moral perception” in the very loose sense of being perceptually aware of a concrete action and experiencing this action in an evaluative manner. I leave it open whether the evaluative experience is sui generis or reducible to the perceptual experience. I address this topic at the end of this section.

9 However, several authors share my phenomenological main idea. Jennifer Church, for instance, argues that “moral perception is immediate in the same way that our perceptions of tables and chairs is immediate […] because its justification is likewise built into its phenomenology” (Church 2013, 191). This is precisely the approach I seek to promote. The difference is that Church does not classify the phenomenology of moral perception as an evaluative phenomenology.

10 Robert Audi also stresses this phenomenological similarity in his careful reflections on moral perception (cf. Audi 2013, p. 160). Cf. particularly Michael Milona’s excellent article in which he seems to agree that due to this phenomenological similarity evaluative experiences should be considered sui generis experiences (Milona 2018).

11 Concerning the relationship between perceptual and evaluative experiences, it seems promising to follow Husserl’s account according to which evaluative experiences are founded (“fundiert”) upon or integrated into “objectifying” acts such as perceptual or imaginative experiences (Husserl 1988, 2004). For insightful phenomenological reflections on the intentional structure of evaluative experiences, (cf. Drummond 2013; Mulligan 2010; Rinofner-Kreidl 2013, 2015).

12 To be sure, there are scholars such as Cappelen (2012), Deutsch (2010), and Ichikawa (2014) who explicitly dispute the claim that intuitions are essential for philosophical inquiry. However, these authors are clearly in the minority.

13 In particular, I agree with Madison 2017 that justification is an intrinsic, irreducible epistemic value.
